# Reliability Analysis and Optimization of Power Terminal Solder Joints in PPS-Packaged IPMs

**DOI:** 10.3390/mi17060749

**Published:** 2026-06-21

**Authors:** Jun Xu, Bin Zhang

**Affiliations:** 1 School of Software Engineering, Xi’an Jiaotong University, Xi’an 710049, China; bzhang82@xjtu.edu.cn; 2 Xi’an Microelectronics Technology Institute, Xi’an 710065, China

**Keywords:** intelligent power module (IPM), power terminal solder joint, reliability, thermal fatigue, deformation

## Abstract

This study investigates the reliability of power-terminal solder joints in intelligent power modules (IPMs) subjected to thermal cycling, random vibration, and packaging/assembly-induced deformation. Fifty IPMs were tested under temperature cycling from −55 °C to 125 °C and random vibration from 20 to 2000 Hz, and the experimental observations were combined with finite element simulations of thermal, vibration, and deformation loads. The modules survived 200 temperature cycles in the free state, whereas functional abnormalities occurred after board-level assembly and subsequent environmental loading. Simulation results showed that random vibration produced limited solder-layer stress because the first structural mode was above the excitation range, while packaging and PCB deformation markedly increased the initial stress of the power-terminal solder joints. When local deformation reached approximately 0.5 mm, the calculated solder-pad stress reached or exceeded the shear-strength risk range, consistent with the failure tendency observed in highly deformed modules. Weibull analysis further indicated a fatigue-dominated failure process with an increasing failure rate. These findings suggest that deformation control, package stiffness improvement, and assembly flatness management are critical for improving the reliability of IPM power-terminal solder joints.

## 1. Introduction

Aerospace technology is rapidly advancing towards electrification, high voltage, and high power density [1,2]. The performance of Insulated Gate Bipolar Transistors (IGBTs) therefore directly determines the efficiency, power density, and reliability of entire electrical systems [3,4]. Intelligent Power Modules (IPMs), as intelligent IGBT modules, integrate power switching chips, drivers, and protection circuits (undervoltage, overcurrent, short-circuit) with high density. Widely used in high-voltage (1.5 kV), high-power applications such as new energy vehicles, industrial servo drives, and smart grids, their reliability directly impacts the lifespan and operational safety of the complete equipment [5,6,7,8]. With the rapid development of electronic technology, the power density and integration level of IPMs are increasing rapidly, making the packaging process critical. The output power terminal, serving as the key electrical connection between the internal power driver chip and the external main circuit, is responsible for conducting high currents and dissipating heat from the chip. Its soldering reliability directly affects the overall reliability of the IPM. During actual operation, IPMs endure severe electro-thermo-mechanical multi-physics field coupled loads.

With the application of wide-bandgap semiconductors like Silicon Carbide (SiC), the power density of IPMs has significantly increased, leading to exacerbated junction temperature fluctuations. This makes solder joints more prone to thermal fatigue crack initiation under conditions of severe thermal expansion coefficient mismatch between upper and lower layermaterials [9,10,11]. Brittle intermetallic compounds (IMCs) continuously grow at the interface between the solder and copper within the IPM during operation. The increase in thickness and coarsening of their morphology significantly reduce interfacial toughness, promoting rapid crack propagation along the IMC layer. This results in a sharp increase in contact resistance and local thermal runaway, ultimately leading to device performance degradation or functional failure [12,13,14,15,16,17,18]. Zhang et al. [19] dynamically observed the IMC evolution and fracture mechanism at Sn2.5Ag0.7Cu0.1RE/Cu lead-free solder joint interfaces, noting a transition in fracture mechanism from ductile to brittle, with the fracture path shifting from the solder seam to the interfacial IMC layer. Other researchers have also conducted relevant studies on the effects of different solder materials on thermal performance, wettability, interfacial microstructure, and soldering performance [20,21,22,23,24].

Inadequate control of IPM soldering materials and processes can lead to increased voiding in solder joints. Industry experience suggests that the total area of all voids in a single chip’s solder layer should not exceed 5% of the chip area, and the maximum single void area should not exceed 2.5% [25]. Chen et al. studied the aging interfacial structure and anomalous tensile strength of SnAg3Cu0.5/Cu solder joints [26]. Li et al. and other researchers conducted comparative studies on the impurity effects on SnAgCu and SnZn solder joints with electrodeposited copper [27,28,29], exploring the impurity effects in joints formed by connecting different solder alloys with electroplated copper layers. Results indicated that impurities significantly affect the interfacial reaction between SAC305 and copper. Numerous voids formed at the interface of high-impurity SAC305/copper, and the growth rate of Cu6Sn5 IMC was faster. Yang et al. [30] investigated the failure mechanism involving creep–fatigue interaction in solder layers under Power Cycling Tests (PCTs). Results showed that initial voids in the solder layer continuously collapsed, while new voids grew. Wei et al. [31] proposed a temperature prediction model for IGBT power semiconductor modules, which can predict localized hot spots and increased junction temperature during operation. The proposed model demonstrates an excellent goodness of fit, with a coefficient of determination of 99.49% on the test set. Dhyani et al. [32] conducted a cross-comparison study of thermal cycling and high-temperature stress on I/O connection elements, finding that changes in package pins under temperature cycling conditions were primarily due to thermo-mechanical damage caused by diffusion and creep. Therefore, under longer heating times and higher temperatures, solder layer failure is considered a complex process involving creep–fatigue interaction [33]. Although solder fatigue and interfacial degradation in IPMs have been widely investigated, most studies have focused on die-attach layers, bond wires, void evolution, and power-cycling-induced degradation. In contrast, the reliability of power-terminal solder joints under packaging deformation and board-level assembly constraints has received less attention. This issue is particularly important for PPS-packaged IPMs, in which package deformation may introduce considerable initial stress before thermal or vibration loading occurs.

As reported by Wei et al. [31], void characteristics (size, location, and percentage) significantly affect the IGBT junction temperature. Although our current simulation assumes void-free solder, we acknowledge that in practice, void percentages of 5–20% are common. Such voids can reduce effective thermal conductivity and accelerate solder fatigue, which will be investigated in our future experimental work.

In summary, this study focuses on the solder joints of power terminals in IPMs. Experimental tests were conducted on 50 IPMs, including temperature cycling from −55 °C to 125 °C, random vibration from 20 Hz to 2000 Hz, and validation of packaging/assembly-induced deformation. The results show that the IPMs survived 200 temperature cycles in the free state, but functional anomalies occurred after board-level assembly and subsequent environmental loading. Finite element simulations indicate that random vibration produces limited stress on the solder layer because the first structural mode is above the excitation frequency range, whereas packaging and PCB deformation markedly increase the initial stress of the power-terminal solder joints. When the local deformation approaches the order of 0.5 mm, the computed solder-pad stress reaches or exceeds the risk range of shear strength, which is consistent with the failure trend observed in highly deformed modules. Weibull analysis further reveals a fatigue-dominated failure process with an increasing failure rate. These findings suggest that deformation control, improvement inpackage stiffness, and management of assembly flatness are critical for enhancing the reliability of power-terminal solder joints in IPMs.

## 2. Materials and Methods

This study adopts a combined experimental and finite element simulation approach.

### 2.1. Thermal Fatigue Experiment and Random Vibration Experiment Design

**Figure 1:**
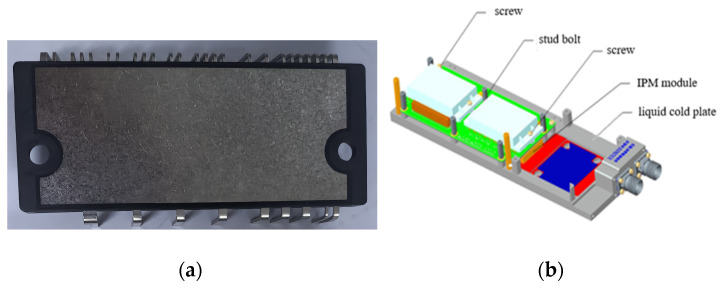
Module Installation Diagram. (**a**) IPM, (**b**) IPM on the PCB.

IPMs, as intelligent IGBT modules, are widely used in high-power applications such as new energy vehicles, industrial servo drives, aerospace electric fuel pumps, starter/generator systems, and electro-mechanical actuation systems. The application model for the IPM used in this study is shown in Figure 1. The overall structure utilizes liquid cooling. The IPM’s printed circuit board (PCB) is fixed onto the liquid cooling plate body using six M3 mounting studs and two M3 screws. The module itself is connected to the PCB via two screws and terminal soldering, ensuring a tight connection between the module and the cold plate. In practical applications, IPMs frequently experience severe temperature fluctuations and vibration environments due to self-heating and environmental changes. These fluctuations can affect the module’s physical structure and electrical performance, thereby impacting its reliability and service life.

In this study, thermal cycling fatigue experiments were conducted to investigate the material stress changes and electrical parameter variations in the high-current power terminal solder joints of IPMs when repeatedly exposed to high- and low-temperature environments. The principle involves setting up a series of temperature cycles to simulate the temperature conditions (from −55 °C to +125 °C) that an IPM might encounter in real-world applications. Through repeated heating and cooling processes, the module is powered on after the experiment to determine changes in its electrical parameters. Temperature cycling experiments typically consist of three phases: temperature transition phase, high-temperature soak phase, and low-temperature soak phase. The design of this experiment referred to the standard: MIL-STD-883J [34], Environmental Test Part 2: Test Methods, Temperature Cycling (Method 1010.8) (See Table 1). The equipment used was a rapid temperature change test chamber. Based on the standard and the actual heating/cooling rate of the chamber, the cycle period was set to one hour, with a temperature transition phase of 1 min and soak times of 30 min at both high and low temperatures. The number of temperature cycles was 200 cycles at the module level, followed by 20 cycles after PCB assembly, with subsequent cycling continuing up to 700 cycles. During the experiment, the static parameters of the IPM were periodically tested and analyzed.

**Figure 2:**
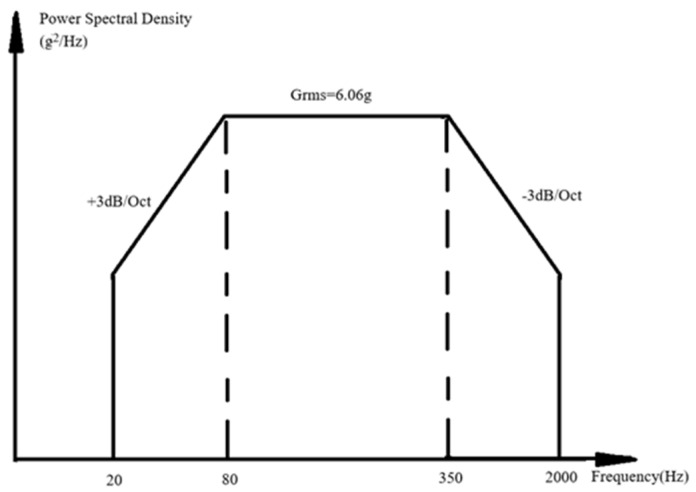
Random Vibration Profile.

This study applies random vibration stress to verify the material stress changes and electrical parameter variations in the IPM’s packaging structure under vibration environments. The principle involves setting up a series of random vibration tests to simulate the vibration conditions an IPM might encounter in practice. The random vibration stress profile follows Test Method 2026 (Random Vibration) as specified in MIL-STD-883J. The experiment used Condition I (E) (See Table 2 and Figure 2), applied sequentially in the X, Y, and Z axes for 15 min per axis. Upon completion of the test, the module’s performance and structural integrity were analyzed: electrical performance tests were conducted, followed by dissection and analysis of the specimens.

### 2.2. Simulation Modeling

**Figure 3:**
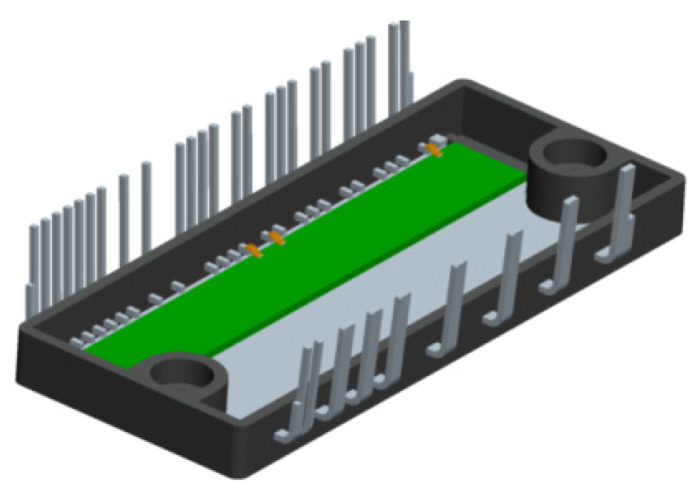
3D Simulation Model of IPM.

This study focuses on the high-current power terminal solder joint of the IPM. Its typical structure involves connecting a copper terminal and the DBC substrate copper layer using Sn-Ag-Cu solder. The primary failure mode under power and temperature cycling loads is fatigue crack initiation and propagation in the solder due to plastic strain accumulation, ultimately leading to electrical connection failure. Additionally, brittle intermetallic compounds form at the solder–copper interface at high temperatures; excessive growth reduces interfacial toughness, accelerating crack propagation along the interface. A 3D simulation model of the IPM was established, as shown in Figure 3. The model includes main components such as the housing, lid, leads, substrate, silver–copper bracket, and solder. After model creation, analyses of the single module’s baseplate stress, thermal stress, and vibration stress were performed. The simulation results were then compared and analyzed against experimental results.

In the IPM, the housing and lid material is PPS, the lead material is Cu, the PCB material is FR4, the baseplate material consists of Cu and Si_3_N_4_, and the bracket material is AgCu45. The material parameters are defined according to the structure’s materials. Although the IPM operates over a wide temperature range, ignoring the temperature dependence of material properties may introduce some inaccuracies—particularly for PPS, whose properties vary more noticeably with temperature. Nevertheless, to maintain a simplified simulation approach, room-temperature material parameters are used, as they still capture the overall trends consistently. Consequently, temperature-independent parameters are employed in this study, as listed in Table 3.

**Figure 4:**
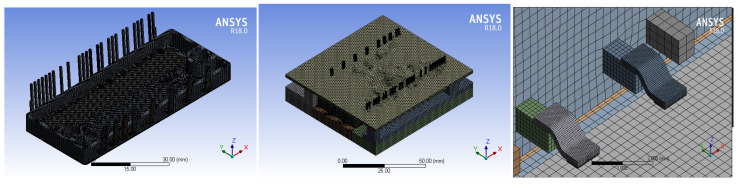
Finite Element Model of IPM.

Based on the various structural components of the model, meshing was performed separately for the case, lid, leads, substrate, silver–copper bracket, and solder to obtain a complete finite element model. To eliminate the influence of discretization error on the stress and strain results of the solder joints, three mesh models with different densities were designed. The maximum equivalent stress was selected as the global quantity, and the equivalent plastic strain at the central solder joint was selected as the local quantity for convergence assessment. From the medium mesh to the fine mesh, the change in the maximum equivalent stress did not exceed the convergence threshold of 5%. Therefore, the medium mesh was adopted to balance computational accuracy and efficiency. The final model consists of 455,113 solid elements and 615,938 nodes, as shown in Figure 4.

## 3. Experimental Results

According to the module production batches, a total of 50 modules from two batches were selected for the experiment, with 24 modules in the first batch and 26 modules in the second batch. Assembly was performed without additional flatness selection to preserve the natural distribution of package deformation. The deformation measurement method used a plane defined by three vertices on the top of the IPM package as a reference, and measured the deviation height of the remaining vertex. The measurement equipment was an optical three-coordinate height gauge. The package deviation data ranged from 36 μm to 321 μm. Two modules were soldered onto each PCB, resulting in a total of 25 PCBs.

**Figure 5:**
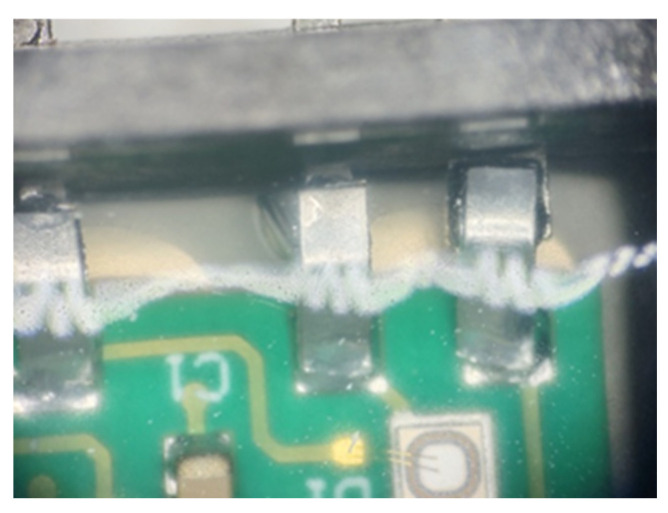
Morphology of the power-terminal leads after temperature cycling.

Stage 1: 50 modules, unassembled state, −55 °C to 125 °C, 200 cycles, 0 failures. After the test, all modules passed functional testing, and no significant creep was observed on the solder joints. Figure 5 shows the morphology of the power-terminal leads after temperature cycling.

Stage 2: After assembly onto PCB and cold plate, 20 cycles followed by random vibration; 1 module (module #17) exhibited functional anomalies.

Stage 3: Continued temperature cycling to 700 cycles; a total of 13 power terminals across 6 modules experienced functional failure (modules #6, #14, #17, #34, #38 and #45).

Stage 4: Module #17 was selected for decapsulation, potting removal, EDS, and cross-section analysis.

**Figure 6:**
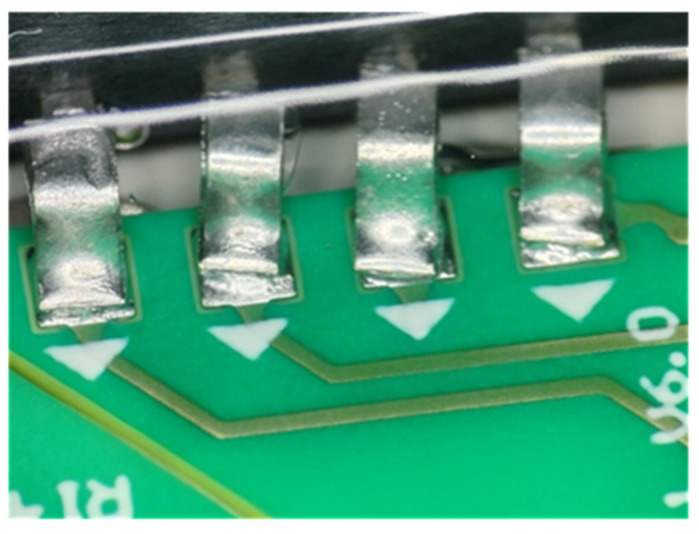
Internal morphology of module #17.

**Figure 7:**
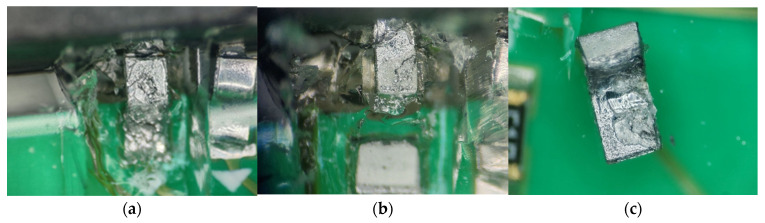
Morphology of module #17 after de-potting. (**a**) Surface morphology of pin 19, (**b**) morphology of pin 19 after peeling, (**c**) morphology of the frame soldering surface.

**Figure 8:**
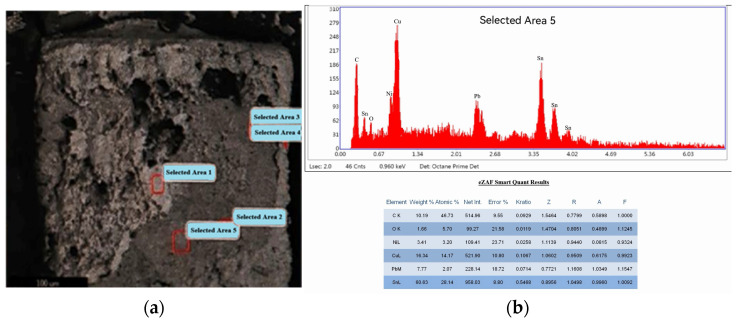
EDS analysis of pin 19 of module #17 after peeling. (**a**) EDS analysis of pin 19 of module #17, (**b**) eZAF Smart Quant Results.

Failure analysis was performed on module #17. The module was decapped and inspected under a microscope; the specific morphology is shown in Figure 6. The failed terminal showed interfacial separation associated with the metallized terminal region rather than poor solder wetting (Figure 7). The presence of Sn and Pb on the interface indicated sufficient wetting of the Ni-plated terminal surface, whereas the observed separation between the Cu substrate and Ni plating suggested that deformation-induced stress may have promoted interfacial delamination in the terminal metallization/solder-joint system (Figure 8).

**Figure 9:**
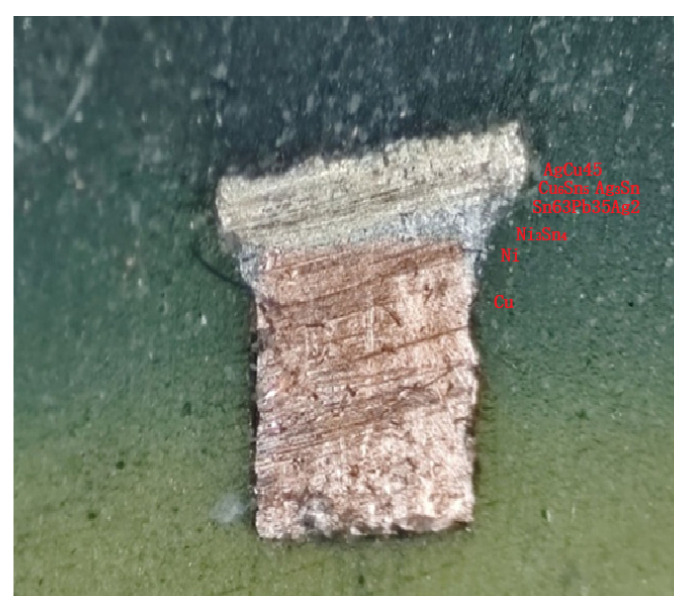
Cross-sectional morphology of the alloy layer of a normal solder joint.

Cross-section samples were prepared from the normal solder joints of the failed module, and the cross-sectional morphology of the alloy layer is shown in Figure 9. It can be seen from Figure 9 that the contact between the frame and the pin is intact without any delamination. The cross-sectional morphology shows that the bonding interface between the frame and the pin is smooth and continuous, with no observed defects such as delamination, voids, or cracks, indicating good bonding strength. This demonstrates excellent adhesion and structural stability between the two components.

## 4. Simulation Results

### 4.1. IPM Stress Analysis Under Temperature Cycling

**Figure 10:**
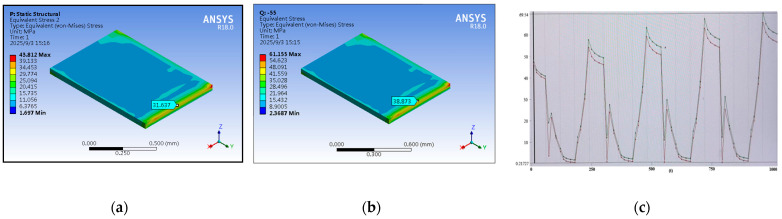
Stress curve of the pad during temperature cycling. (**a**) Solder Pad Stress Contour at High Temperature 125 °C, (**b**) Solder Pad Stress Cloud Image at Low Temperature −55 °C, (**c**) stress curve of the pad during temperature cycling.

Sn63Pb35Ag2 is a lead-containing solder alloy based on the traditional Sn63Pb37 eutectic solder, with the addition of approximately 2 wt% silver (Ag). The introduction of Ag significantly improves the mechanical properties and microstructure of the solder, enabling it to retain application value in electronic packaging fields with stringent reliability requirements. The addition of Ag exerts two effects on the Sn63Pb37 matrix: first, the formation of dispersed Ag_3_Sn intermetallic compound particles; second, the enhancement of matrix strength through solid solution strengthening. These dispersed Ag_3_Sn particles, distributed along grain boundaries and within grains, effectively hinder dislocation motion and grain boundary sliding, thereby improving the material’s creep resistance and thermal fatigue resistance, and effectively enhancing the overall mechanical properties of the material. In this paper, the Anand unified viscoplastic constitutive model is used for description.The Anand parameters were taken as the built-in default parameters in the ANSYS R18.0 software. This model employs an internal variable (deformation resistance) to characterize the evolution of the internal dislocation network without explicitly distinguishing between plasticity and creep, making it suitable for stress–strain calculations of solder joints under temperature cycling loads. The thermal stress magnitude of the board-level pad is shown in Figure 10.

According to the simulation analysis results, when the module experiences drastic high–low temperature changes, the stress on the terminal solder joints undergoes severe fluctuations during the temperature variation process. This also indicates that temperature cycling has a significant impact on the reliability of the solder joints. The temperature swing induced repeated stress fluctuations in the terminal solder joints, indicating the possibility of fatigue damage accumulation during cycling. However, a cycle-by-cycle damage model is required to quantitatively predict fatigue life.

### 4.2. IPM Random Vibration Stress Analysis

**Figure 11:**
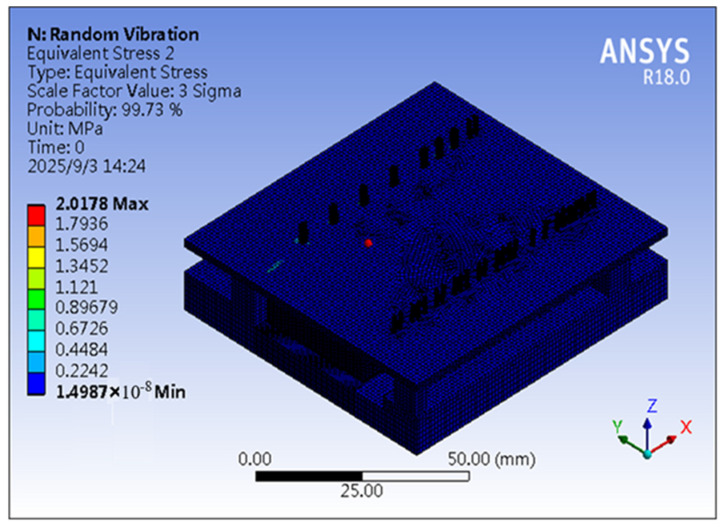
Structural Stress Contour under Random Vibration.

Constraints were applied to the assembled IPM, and a modal analysis was performed. The resulting frequency results are shown in Table 4. Vibration stress was applied to the IPM, yielding the structural stress contour shown in Figure 11. According to the simulation analysis results, under vibration stress conditions, the calculated stress in the terminal solder region remained limited, having almost no impact on the solder layer.The first simulated natural frequency of the assembled module was 2261.2 Hz, which was higher than the upper frequency of the random vibration profile. This frequency separation partly explains the limited solder-joint stress response under the specified vibration condition.

### 4.3. IPM Installation Stress Analysis

Since the module packaging material is PPS, significant packaging deformation occurs during the installation process. Therefore, corresponding stress analysis is performed.

(1) Substrate Deformation (Convex Shape)

**Figure 12:**
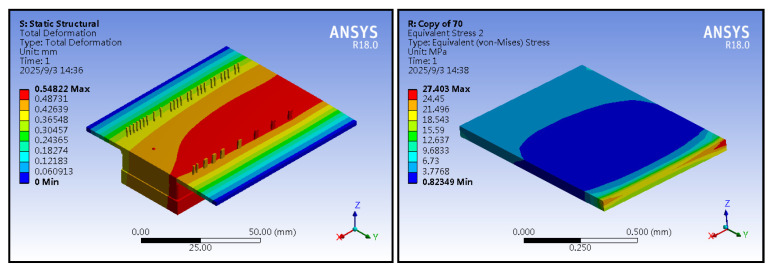
PCB Deformation Contour. (Convex deformation, local displacement = 0.38 mm, solder stress ≈ 18 MPa).

The assembly stress experienced by the module after being mounted onto the PCB is greater than the stress of the module in the free state. Under the conditions of applying fixed constraints to both sides of the PCB and applying an upward force at the bottom of the structure, the stress distribution contour of the structure was obtained, as shown in Figure 12.

According to the simulation analysis results, when the overall deformation of the PCB reaches 0.55 mm, the deformation of the PCB at the fine lead locations is approximately 0.38 mm. At this point, the stress on the solder reaches its maximum, primarily concentrated at the central lead position. Excluding stress concentration effects, the stress on the solder pad is about 18 MPa, indicating that the normal pads are reliable and at no risk of fracture (Figure 12).

**Figure 13:**
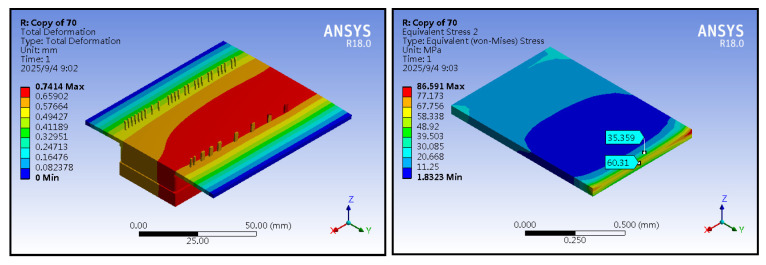
PCB Deformation Contour.(Convex deformation, local displacement = 0.55 mm, solder stress ≈ 60 MPa).

According to the simulation analysis results, when the overall deformation of the PCB reaches 0.75 mm, the deformation of the PCB at the fine lead locations is approximately 0.55 mm. The stress on the solder reaches its maximum at the central lead position. Excluding stress concentration effects, the stress on the solder pad is about 60 MPa, exceeding the maximum shear strength of a normal pad, indicating a high risk of failure (Figure 13).

(2) Substrate Deformation (Concave Shape)

Constraints were applied to both sides of the PCB to simulate its boundary conditions in an actual installed state. Simultaneously, a vertically downward concentrated force was applied at the bottom of the structure to simulate the effect on the structure when the PCB warps upwards in the middle. The stress distribution under this load condition is shown below.

**Figure 14:**
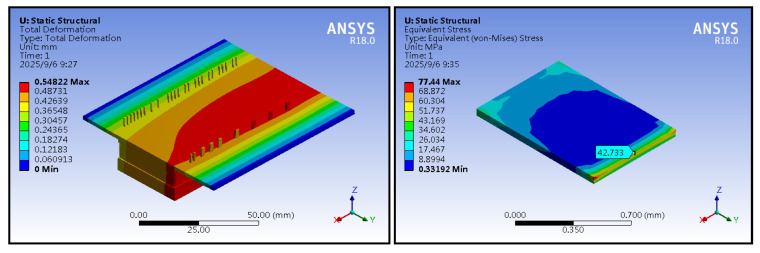
PCB Deformation Contour.(Concave deformation, local displacement = 0.39 mm, solder stress ≈ 42 MPa).

According to the simulation analysis results, when the overall deformation of the PCB reaches 0.55 mm, the deformation of the PCB at the fine lead locations is approximately 0.39 mm. The stress on the solder reaches its maximum at the central lead position. Excluding stress concentration effects, the stress on the solder pad is about 42 MPa (Figure 14).

**Figure 15:**
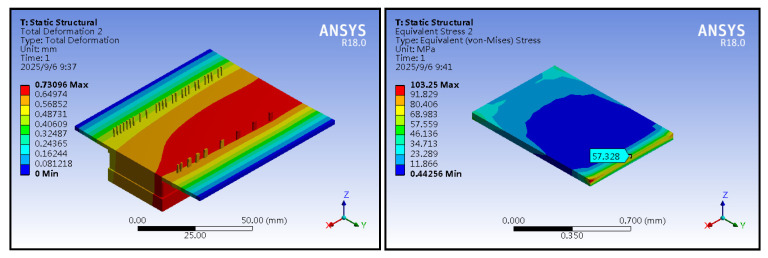
PCB Deformation Contour.(Convex deformation, local displacement = 0.54 mm, solder stress ≈ 57 MPa).

According to the simulation analysis results, when the overall deformation of the PCB reaches 0.73 mm, the PCB deformation at the fine lead positions is approximately 0.54 mm. At this point, the stress on the solder is at its maximum, primarily concentrated at the center lead position. After eliminating the stress concentration effect, the stress at the solder pad is about 57 MPa, which exceeds the maximum shear strength of a normal pad, indicating a high risk of failure (Figure 15).

Table 5 presents a comparison of solder-joint stress under different loading conditions.

## 5. Coupled Failure Mechanism and Life Analysis and Discussion

Based on the experimental validation and simulation analysis data described above, the reliability of the terminal solder joints of IPMs under actual installation conditions after temperature cycling and vibration tests is analyzed and discussed. The temperature cycling test conditions and vibration stress conditions were set with reference to the MIL-STD-883J standard. Since the temperature cycling failure mechanism of solder joints is already known, further analysis and discussion on this mechanism are not provided in this paper; only the experimental results and simulation analysis results obtained in this study are discussed.

### 5.1. Rationality Analysis of Boundary Conditions

During the actual IPM packaging and installation process, the terminal solder joints connect the chip and the package housing. The entire IPM is supported and constrained by the PCB. In the simulation model, constraints were applied to both sides of the PCB to simulate the boundary conditions under actual installation conditions. Since the IPM packaging material is PPS, whose elastic modulus is nearly an order of magnitude lower than that of the chip, terminals, etc., the package housing was not simplified. In addition, a sensitivity test was conducted on the fixed boundary condition. The PCB support constraint was changed to an elastic support (with stiffness values of 0.5 times and 2 times the original value, respectively). The resulting changes in solder joint stress did not exceed 10%, indicating that the fixed boundary assumption has a limited effect on the results.

### 5.2. Quantitative Comparison Between Simulation and Experiment

To comprehensively validate the effectiveness of the simulation model, quantitative comparisons were performed from three perspectives: temperature cycling stress response, vibration stress response, and installation deformation response.

**Figure 16:**
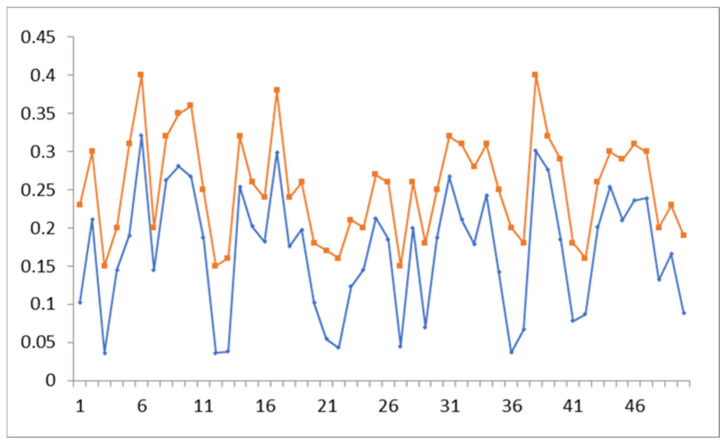
Deformation of modules before and after assembly. Symbols: ◊ (before assembly) and ▪ (after assembly). The blue and orange lines represent the spread of deformation before and after assembly, respectively.

In the actual experiments, a total of 50 IPMs in two groups were tested. First, the deformation of the IPM itself and the deformation after mounting onto the PCB were measured, as shown in Figure 16. From the actual experimental results, one module in each group failed, namely #17 and #38. In terms of post-installation deformation, these two modules exhibited relatively large deformation among the 50 modules. The simulation data showed that when the module deformation exceeded 0.5 mm, the stress on the terminal solder joints presented a risk of failure, which is consistent with the trend observed in the actual experimental results.

Vibration stress response: Through simulation and experimental verification, due to the relatively large size of the power terminal solder joints of the IPM, when vibration stress is applied solely to the power terminals of the IPM, the stress on the IPM is very small, and its impact on the solder layer is limited.

Temperature cycling strain response: According to the simulation analysis results, when the module experiences drastic high–low temperature changes, the stress on the terminal solder joints undergoes severe fluctuations during the temperature variation process. This also indicates that temperature cycling has a significant impact on the reliability of the solder joints. Comparative analysis of the actual experimental results shows that the IPMs underwent 200 cycles in the free state without any failure. However, after being mounted onto the PCB and subjected to 20 temperature cycles, one failure occurred in each of the two experimental groups. This indicates that under conditions of significant initial deformation, the ability of the IPM terminal solder joints to withstand temperature cycling stress decreases dramatically.

### 5.3. Weibull Life Distribution Analysis

**Figure 17:**
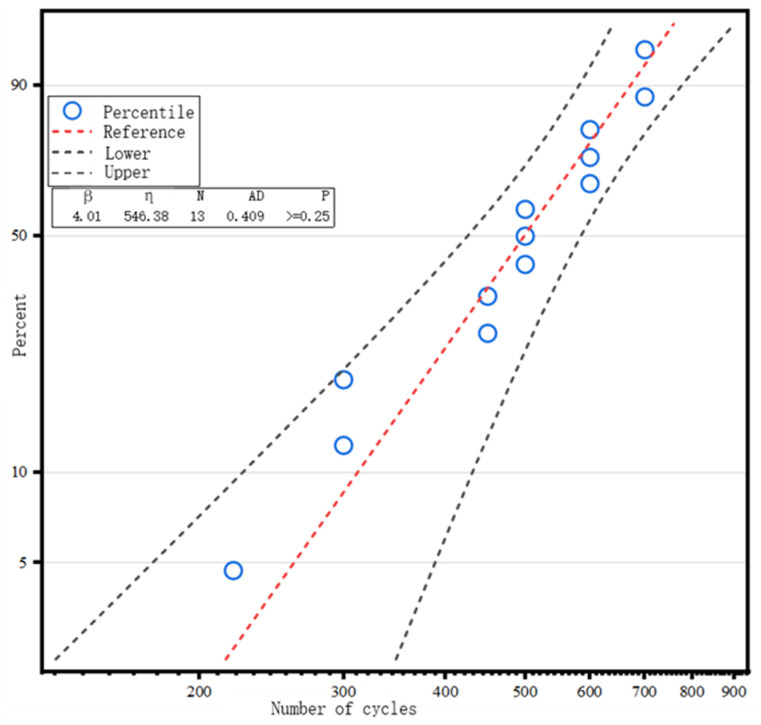
Weibull life distribution.

A total of 50 IPM samples were tested, among which 13 power-terminal solder joints failed (N presents the failed power-terminal solder joints). The failure life data were described using a two-parameter Weibull distribution. The shape parameter β and scale parameter η were estimated by median rank regression. As shown in Figure 17, β = 4.01, η = 546.38 cycles (N = 13 N presents the failed power-terminal solder joints). The Anderson–Darling statistic = 0.409, P > 0.25, indicating a good fit to the Weibull distribution. β > 1 indicates that the failure rate increases with the number of cycles, which is consistent with the characteristics of fatigue wear. Because not all modules failed by the end of 700 cycles, the non-failed samples were treated as right-censored observations in the Weibull analysis.

Through the above experimental verification and simulation analysis, the following conclusions can be drawn:

(1) Since the packaging material of the IPM is PPS, whose strength is much lower than that of ceramic or metal packaging, when the packaging deformation is not controlled during the production process, the module itself undergoes significant deformation, which can exceed ±0.3 mm at maximum. After being mounted onto the PCB, the deformation of the IPM becomes even larger, reaching up to ±0.4 mm. Simulation and experimental verification show that when the packaging deformation of the IPM is large, the initial stress at the internal power terminals increases significantly, reaching or exceeding the ultimate shear strength of normal solder joints, posing a high risk of failure. Subsequently, under the combined effects of temperature cycling stress and vibration stress, the terminal solder joints ultimately fail.
(2) Unlike traditional chip solder layer or bond wire failures, the terminal solder joint failures discussed in this paper are more significantly affected by assembly geometric deformation and packaging stiffness.
(3) Temperature cycling is a necessary environment for fatigue accumulation, but the initial deformation determines the stress baseline of the solder joints before cycling.
(4) Random vibration has a weak contribution under the experimental conditions of this study, but this does not imply that vibration is unimportant in all structures; when the structural modal frequencies fall within the excitation frequency range or the terminal size decreases, the vibration risk may increase.
(5) The PPS packaging material has relatively low stiffness; if flatness control is insufficient, it will amplify the local terminal stress under the constraint of the PCB.

These results complement previous studies that mainly attributed solder-joint degradation in power modules to thermal fatigue, void evolution, and intermetallic compound growth. In the present IPM configuration, packaging and assembly deformation acted as an initial-stress amplifier, reducing the reliability margin of the power-terminal solder joints before subsequent thermal cycling.

Several limitations should be noted. First, the experimental samples were based on one IPM package configuration and one terminal solder-joint design; therefore, the quantitative deformation thresholds may vary with package size, terminal geometry, solder alloy, and substrate structure. Second, the finite element model used material parameters obtained from the literature or standard databases, and the temperature dependence and aging evolution of some material properties were simplified. Third, the vibration conclusion is specific to the tested random vibration spectrum and the simulated modal characteristics of the assembled structure. Finally, the Weibull analysis was based on limited failure events, and additional samples or longer cycling tests would be useful for refining the life prediction model.

## 6. Design Implications

Based on experimental data and simulation results, the following design guidelines can be provided.

(1) Enhance the intrinsic strength of the IPM packaging material. Replace the PPS material with reinforced PPS containing 20% glass fiber. The tensile strength can be doubled from approximately 55 MPa for pure PPS to 110 MPa. The reinforced PPS also exhibits excellent creep resistance and fatigue resistance. Although the cost of reinforced PPS is higher, it offers better creep resistance, fatigue resistance, and coefficient of thermal expansion, and its packaging process is fully compatible with the original packaging.
(2) By adopting reinforced PPS material and optimizing the IPM cover design, such as adding reinforcing ribs, the encapsulation deformation of the IPM can be effectively controlled. To effectively reduce the risk, the recommended deformation control target for the IPM encapsulation is ≤±0.1 mm.
(3) When mounting the module, the total mounting deformation of the PCB control target is recommended to be ≤±0.2 mm. When total mounting deformation exceeds approximately 0.5 mm, local stress approaches or enters the high risk zone.

## 7. Conclusions

This paper employs a combined approach of simulation and experimentation, including simulation analysis, temperature cycling tests, vibration stress tests, and simulation analysis under packaging deformation stress conditions. This approach verifies the internal stress distribution characteristics and stress mechanisms of IPM power terminal solder joints under multiple stress conditions.Based on experimental and simulation analyses, we draw the following conclusions:

(1) The IPMs survived 200 temperature cycles in the free state, indicating that thermal cycling alone did not immediately trigger terminal solder-joint failure under the tested condition.
(2) Functional abnormalities occurred after board-level assembly and subsequent environmental loading, and the failed modules exhibited relatively large post-assembly deformation, suggesting that assembly-induced deformation reduced the reliability margin of the terminal solder joints.
(3) Finite element results showed that random vibration produced limited stress in the power-terminal solder region under the specified 20–2000 Hz profile, whereas packaging/PCB deformation generated much higher local stress.
(4) When local deformation reached approximately 0.5 mm, the calculated solder-pad stress reached the high-risk range, which was consistent with the failure tendency observed in the experimental samples.
(5) Deformation control, improved package stiffness, and stricter assembly flatness management are therefore recommended as primary measures for improving the reliability of IPM power-terminal solder joints.

## Figures and Tables

**Table 1:** Temperature Cycling Test Conditions.

Test Condition	Temperature Range	Number of Cycles	Typical Application
Condition B (Referred)	−55 °C~+125 °C	200 cycles at module level, 20 cycles after board-level assembly, and then continued until a total of 700 cycles.	General screening condition, transition time < 1 min, soak 30 min at extremes.

**Table 2:** Vibration Test Conditions.

Test Condition	Parameter	Requirement
Condition I	Frequency Range	20~2000 Hz
	Power Spectral Density (PSD)	0.04 g^2^/Hz
	Acceleration (Grms)	7.56 Grms
	Test Axes	Vibration applied sequentially in three mutually perpendicular axes (X, Y, Z)
	Duration	15 min per axis

**Table 3:** Material Parameters of IPM.

Material	Density (kg/m^3^)	Elastic Modulus (GPa)	Poisson’s Ratio	CTE (ppm/°C)
PPS	1350	3.75	0.39	50
PCB	1450	25	0.15	60
Cu	8900	108	0.32	16.5
Si_3_N_4_	3210	250	0.25	3
AgCu45	9900	83	0.35	19.6
Pb63Sn35Ag2	8440	29.9	0.35	27
Aluminum Alloy	2770	71	0.33	23

**Table 4:** Structural Modes and Frequencies.

Mode	1st	2nd	3rd	4th	5th	6th
Frequency/Hz	2261.2	2783.8	2927.5	3490.4	4068.3	4365.4

**Table 5:** Comparison of solder-joint stress under different loading conditions.

Load Case	Key Input	Representative/Max Solder Joint Stress	Failure Risk Assessment	Implication
Temperature cycling	−55 °C to 125 °C	30/38 Mpa	Moderate/cumulative risk	Thermal fatigue is a long-term degradation factor
Random vibration	20–2000 Hz, 7.56 Grms	1/2 Mpa	Low	First mode is above excitation upper limit, vibration contribution is limited
Convex assembly deformation	Local deformation approx. 0.38/0.55 mm	35/60 MPa	High risk near 0.55 mm	Deformation significantly increases initial stress
Concave assembly deformation	Local deformation approx. 0.39/0.54 mm	42/57 MPa	High risk	Concave deformation may also induce terminal failure

## Data Availability

The original contributions presented in this study are included in the article.
